# MDSCs: Key Criminals of Tumor Pre-metastatic Niche Formation

**DOI:** 10.3389/fimmu.2019.00172

**Published:** 2019-02-07

**Authors:** Yungang Wang, Yanxia Ding, Naizhou Guo, Shengjun Wang

**Affiliations:** ^1^Department of Laboratory Medicine, The First People's Hospital of Yancheng City, Yancheng, China; ^2^Department of Laboratory Medicine, The Affiliated People's Hospital, Jiangsu University, Zhenjiang, China; ^3^Jiangsu Key Laboratory of Laboratory Medicine, Department of Immunology, School of Medicine, Jiangsu University, Zhenjiang, China; ^4^Department of Dermatology, The First People's Hospital of Yancheng City, Yancheng, China

**Keywords:** metastasis, pre-metastatic niche, myeloid-derived suppressor cells, formation, evolution, detection

## Abstract

The emergence of disseminated metastases remains the primary cause of mortality in cancer patients. Formation of the pre-metastatic niche (PMN), which precedes the establishment of tumor lesions, is critical for metastases. Bone marrow-derived myeloid cells (BMDCs) are indispensable for PMN formation. Myeloid-derived suppressor cells (MDSCs) are a population of immature myeloid cells that accumulate in patients with cancer and appear in the early PMN. The mechanisms by which MDSCs establish the pre-metastatic microenvironment in distant organs are largely unknown, although MDSCs play an essential role in metastasis. Here, we summarize the key factors associated with the recruitment and activation of MDSCs in the PMN and review the mechanisms by which MDSCs regulate PMN formation and evolution. Finally, we predict the potential value of MDSCs in PMN detection and therapy.

## Introduction

Metastasis remains the leading cause of cancer-related death. Decades of investigations into cancer metastasis have focused largely on the causes of oncogenic transformation and the incipient emergence of tumors, although Stephen Paget proposed the seed-and-soil hypothesis in 1889 (1). Metastasis-related high mortality has driven cancer biologists to renew their focus on the problem of metastasis. The study of how tumor cells lead to metastasis, such as altering the microenvironment, entering the circulation, and colonizing distant organs, has received more attention. Tireless research efforts have revealed that metastasis results from the interplay of wandering tumor cells with a supportive microenvironment in target tissues (2). The theory that a preconditioned microenvironment that receives incoming cancer cells at secondary organs or sites, termed the PMN, is the key determinant of cancer metastasis is widely accepted. Fidler et al. found that although mouse B16 melanoma cells could be found in the vasculature of multiple organs (3), only lung sites consistently developed metastatic tumor deposits, which provided support for this theory. Kaplan's research in 2005 first demonstrated the existence and stepwise progression of the PMN in Lewis lung carcinoma cells (LLC) or B16 cell-bearing mice (4). However, the complex processes and molecular mechanisms involved in PMN formation have remained among the greatest mysteries surrounding cancer metastasis.

This supportive PMN is prepared by resident cells (5), recruited bone marrow-derived cells (BMDCs) (4), soluble factors (6), and extracellular vesicles (EVs) (7, 8). BMDCs are the main cellular components of the PMN, which is initiated by many types of primary tumors, including colorectal cancer (9, 10), breast cancer (11), and melanoma (12). The evidence of BMDCs in PMN formation is primarily drawn from mouse models and largely focused on the lung and liver as a target organ, although other organs and pathological samples from patients have also been examined. Lewis lung carcinoma (LLC) cells and B16 melanoma cells possess a more widely disseminated metastatic potential and tend to metastasize to the lungs and liver. LLC or B16 tumors are more general models for the PMN related research. Rosandra et al. confirmed the role of BMDCs in PMN formation through LLC or B16 tumors (4). In this study, C57BL/6 mice were lethally irradiated and transplanted with GFP^+^ bone marrow cells. Mice were injected intradermally with either LLC or B16 cells. After irradiation, but before tumor implantation, minimal BMDCs were observed in the lungs or liver. After tumor implantation, but before the arrival of tumor cells, the extravasation and cluster formation of BMDCs were detected near distal alveoli and terminal bronchioles, both common sites for future tumor metastasis. Until day 16, tumor cells were detected and more than 95% of tumor cells co-clustered with GFP^+^ BMDCs. Therefore, factors provided by the primary tumor promote BMDCs to mobilize to pre-metastatic sites, and this migration precedes the arrival of tumor cells. However, the mechanisms by which BMDCs mediate the outgrowth of metastatic cancer cells are not completely understood.

Neutrophils can be expanded, mobilized and recruited to the PMN when the primary tumor occurs. However, the role of neutrophils in PMN formation is not consistent. In colorectal cancer model mice, tissue inhibitor of metalloproteinases (TIMP)-1 creates a PMN in the liver through SDF-1/CXCR4-dependent neutrophil recruitment (13). In mouse models of breast cancer, G-CSF-mobilized Ly6G^+^Ly6C^+^ granulocytes home to distant organs before the arrival of tumor cells and produce the Bv8 protein, which stimulates tumor cell migration through activation of prokineticin receptor (PKR)-1 (14). This result is also observed during early breast cancer progression, G-CSF directs the production of T cell-suppressive neutrophils, which preferentially accumulate in peripheral tissues but not in the primary tumor (15). Specifically, tumor-secreted CCL2 stimulates neutrophils to accumulate in the lung prior to the arrival of metastatic cells and inhibits metastatic seeding by generating H_2_O_2_ in breast cancer mice (16). Thus, neutrophils could promote PMN formation. However, another study in breast cancer mice showed that neutrophils kill tumor cells through ROS production and granzyme-B release (17). Therefore, neutrophils can be a double-edged sword in PMN formation. Currently, the markers used to define neutrophils are oversimplified, and these neutrophils cannot actually be distinguished from MDSCs. Therefore, phenotypic analysis of these neutrophils is still a matter of study and deeper immunophenotyping and functional assessment of PMN-infiltrating immune cells are required.

MDSCs are a heterogeneous group of myeloid cells with immunosuppressive properties that are derived from myeloid progenitor cells and immature myeloid cells. MDSCs have been detected in the lungs of mice bearing mammary adenocarcinoma prior to metastatic spread (18). MDSCs have been shown to play pleiotropic roles in cancer progression by shaping the tumor microenvironment and metastatic niches through immunosuppression and inflammation. Expanding experimental evidence indicates that MDSCs are the key determinants of PMN formation, although other immune cells, such as neutrophil, macrophage and Tregs also involved in PMN formation (19). S100A8/A9 imaging shows that MDSCs are abundant in the pre-metastatic lung and correlate with the subsequent metastatic breast cancer burden (20). In breast cancer model mice, MDSCs accumulate in the PMN and suppress cytotoxic CD8^+^ T cells and NK cells through the productions of reactive oxygen species (ROS) and arginase 1 (Arg-1) (21). MDSCs are also involved in an array of non-immunological functions that may be associated with the PMN through secretion of cytokines, chemokine's, growth factors and exosomes. The roles of MDSCs in PMN formation and evolution are diverse and may range from the induction of vascular leakage and extracellular matrix (ECM) remodeling to systemic effects on the immune system that facilitate metastatic outgrowth (19, 22, 23).

In this review, we summarize the new phenotypic features of MDSCs and the main factors that regulate MDSC recruitment and expansion in the PMN. We mainly discuss the multi-faceted superior capacity of MDSCs to establish a pre-metastatic microenvironment in distant organs, and finally provide new insights into how this process can be translated into clinical applications.

## MDSC Phenotype and Function

MDSCs are a heterogeneous population of immature myeloid cells whose numbers are increased in states of cancer, inflammation, or infection (24, 25). At present, most knowledge about MDSCs comes from tumor immunity research. Tumor cells mobilize MDSC differentiation, proliferation, and migration toward tumor tissue by secreting vascular endothelial growth factor (VEGF), granulocyte-macrophage colony stimulating factor (GM-CSF), IL-6, IL-10, transforming growth factor-β (TGF-β) and other factors (26). The heterogeneity of MDSCs is derived from the complex expression patterns of their surface markers and locations. MDSCs mainly include monocytic MDSC (M-MDSC) and granulocytic MDSC (G-MDSC) (or polymorphonuclear MDSC, PMN-MDSC) subpopulations. Other subsets further characterized in human MDSC are the immature MDSC [also known “early-stage MDSC” [eMDSC]] and fibrocytic MDSCs (F-MDSCs). MDSC subsets have been characterized (27), and play critical roles in tumor progression through different mechanisms (Table 1) (32–37). It is worth noting that the dynamic interplay between cancer and host immune system often affects the process of myelopoiesis. The difference of MDSCs locations have contributed to the complex expression patterns of surface markers and effector molecules (Table 1) (28–30). G-MDSCs and M-MDSCs use different mechanisms for immunosuppression, which have been reviewed elsewhere (31, 39, 40). Briefly, G-MDSCs mainly suppress T cell responses by producing ROS (reactive oxygen species) via an antigen-specific approach. M-MDSCs produce high amounts of NO, Arg-1 and immunosuppressive cytokines, such as IL-10, which suppress both antigen-specific and non-specific T cell responses. M-MDSCs have higher suppressive activity than G-MDSCs. F-MDSCs suppress T cell proliferation through IDO (indoleamine oxidase) production and promote Treg cell expansion.

**Table 1:** Phenotype and function of MDSCs.

**Subset**	**Phenotype (Mouse)**	**Phenotype (Human)**
Total MDSC	CD11b^+^Gr-1^+^CD11c^−^F4/80^+/−^CD124^+^	HLA-DR^−^CD11b^+^CD33^+^
G-MDSC	CD11b^+^Gr-1^hi^Ly6C^low^Ly6G^+^CD49d^−^	CD33^+^CD14^−^CD11b^+^CD15^+^(or CD66b^+^)
M-MDSC	CD11b^+^Gr**-**1^mid^Ly6C^hi^Ly6G^−^CD49d^+^	CD11b^+^CD14^+^HLA-DR^low/−^CD15^−^
e-MDSC	–	Lin^−^(CD3/14/15/19/56) HLA-DR^−^CD33^+^
F-MDSC	–	CD11b^low^CD11c^low^CD33^+^ IL-4Ra^+^
**Category**	**Surface molecule (Mouse)**	**Effector molecule (Mouse)**
BM derived progenitors	CD133, CD34, CD117, VLA-4	ROS (28)
MDSCs in BM	CD11b, Ly6G, Ly6C	ROS, Bv8^low^ (29)
MDSCs in blood	CD11b, Ly6G, Ly6C	ROS, Bv8 (29, 30)
Tumor-infiltrating MDSCs	CD11b, Ly6G, Ly6C, CD115, F4/80, CD80	Arg-1, iNOS, NO2^−^, Bv8 (29, 30)
MDSCs in PMN	CD11b, Ly6G, Ly6C^low^, CD62L^low^, CD16^low^	ROS, Arg-1 (18, 31)
**Function**	**Description**	
Immune suppression	Inhibit T-cell proliferation, NK cell and CTL activity, IL-2 production, and promote Treg induction and M2 macrophage reprogramming through secreting Arg-1, ROS, NOS2, IDO, TGF-β, IL-10, and exosomes or membrane molecules (32, 33).	
Tumor angiogenesis	Promote blood vessel formation through upregulating MMP9, VEGF, and Bv8 expression (34, 35).	
Tumor cell stemness	Trigger miR-101 expression and target the CtBP2 (36).	
Metastasis dissemination	Support the epithelial-mesenchymal transition through secreting hepatocyte growth factor and TGF-β1 (37). Regulate resident cell and angiogenesis through exosomal miRNA (38).	

MDSCs are defined based on their phenotypic, functional, and molecular features. Notably, the expression levels of the molecules often change with environmental changes. Primary tumor derived factors mobilize MDSCs from bloodstream into the tissues where metastasis is about to occur. In pre-metastatic lung tissue, MDSCs are indicative of the granulocytic nature of CD11b^+^Ly6C^lo/med^Ly6G^+^ cells which is referred to as G-MDSC or PMN-MDSC. Compared to neutrophils, these cells have fewer granules, diminished CD62L, and CD16 expression. Moreover, these cells express a high level of ROS and Arg-1(31). In mice bearing mammary adenocarcinoma, MDSCs have been detected in the pre-metastatic lung. Phenotypic analysis revealed that Ly6G^+^Ly6C^lo^ cells constituted the major share of such cells, which was indicative of G-MDSCs (18). MDSCs in pre-metastatic tissue provide an microenvironment that is suitable for the arrival and settlement of tumor cells through promoting immunosuppression, leaky vasculature, and collagen restructuring in the premetastatic tissue.

Researchers also define MDSC subpopulations using intrinsic and extrinsic cell death pathway properties, which are involved in myeloid lineage development and survival. The anti-apoptotic molecule cellular Fas-associated death domain-like interleukin-1 β converting enzyme inhibitory protein (c-FLIP) is constitutively required for the development of M-MDSCs, whereas G-MDSCs require a different anti-apoptotic molecule [myeloid cell leukemia 1 (MCL-1)] for development (41). The ability to suppress immune cells is an important standard that is used to define MDSCs. Suppression of T cell activity, including reduced proliferation and suppressed IFN-γ and IL-2 production, is an important standard for the evaluation of MDSC immunosuppressive function (33). The phenotypic and functional characteristics of MDSCs in the PMN need to be further investigated, although current studies have shown that MDSCs in the PMN originate from the bone marrow. In addition, the role of the PMN in MDSC functions and phenotypes is unclear.

## PMN Formation and Evolution

Previous studies investigating tumor metastasis focused largely on identifying cancer cell intrinsic determinants, such as genes and pathways that regulate colonization. Currently, promotion of the spread of tumor cells to secondary organs by prior formation of a supportive PMN at distant sites before the arrival of metastatic cells is widely accepted. The PMN has become a new paradigm for the initiation of metastasis, although understanding the complexity of the PMN is daunting. Indeed, a number of elements are involved in the formation and evolution of the PMN, including cells from different lineages, blood flow, soluble factors, EVs, extracellular matrix, and signaling molecules that can provide niches for tumor settlement and growth. The pathological processes that occur before the development of macrometastases require better understanding. Kaplan's research in 2005 first demonstrated the existence and stepwise progression of the PMN (4). Researchers have uncovered the usual progression of PMN formation in diverse tumors, including colorectal cancer (9, 10), breast cancer (11) and melanoma (12, 42). First, tumor-secreted factors, the effects of surgery, infection and aging not only change blood flow and vascular leakage but also contribute to activation and recruitment of BMDC populations (43, 44). Second, the biological behavior of resident cells changes, and the ECM in the PMN is remodeled (7, 42, 45). Third, a microenvironment with inflammation, immunosuppression, and coagulation disorders is established, which is beneficial for the ability of arriving tumor cells to settle down and survive (19).

## Regulation of MDSC Recruitment and Activation in the PMN

Significant advancements have been made in understanding the regulation of MDSC accumulation and expansion in primary tumors. Currently, diverse factors, including GM-CSF (46), interleukins (47),VEGF (48), tumor-derived molecules (49), prostaglandin E2/cyclooxygenase-2 (PGE2/COX2) (50), EVs (51), complement molecules (52), and IFN-γ (53), have been determined to regulate MDSC accumulation and expansion in the tumor microenvironment through the signal transducers and activators of transcription 1 (STAT1) or STAT3 signaling pathway (54). However, how MDSCs migrate into pre-metastatic sites and become activated is unclear, although research results have shown that MDSCs can infiltrate into the PMN in the presence of soluble factors, including GM-CSF, VEGF, IL-6, IL-1β, and CCL2 (55). The main factors that affect the accumulation and activation of MDSCs in PMN are summarized in Table 2.

**Table 2:** Factors associated with MDSC accumulation/activation in the PMN.

**Molecules**	**Source**	**Receptors**	**Phenotype**	**Model**	**Sites**	**References**
CXCL1	TAMs	CXCR2	CXCR2^+^ MDSCs	Colorectal carcinoma	Liver	(49)
CCL12	Lung	–	M-MDSCs	Melanoma	Lung	(52)
MCP-1/CCL2	BMDCs	CCR2	MDSCs	Skin/Breast cancer	Skin/Lung	(21, 56)
CXCL12	HSCs	CXCR4	MDSCs	Pancreatic tumor	Liver	(57)
CCL15	Colorectal tumor cells	CCR1	CCR1^+^ MDSCs	Colorectal cancer	Liver	(55)
CCL9	G-MDSCs	CCR1	G-MDSCs	Melanoma/Breast cancer	Lung	(54)
Exosomal Hsp72	Tumor cells	TLR2	MDSCs	Colon carcinoma	–	(58)
Exosomal MET	Melanoma	–	MDSCs	Melanoma	Lung	(12)
S100A8/9	MDSCs	TLR4	MDSCs	Breast/ Gastric/ Lung cancer	Lung	(59)
Periostin	MDSCs	–	M/G-MDSCs	Breast tumor	Lung	(60)
ER stress	Neutrophils	–	LOX-1^+^ PMN-MDSCs	HNC NSCLC	Lung	(61)
LOX	Breast tumor cell	–	CD11b^+^ myeloid cells	Breast tumor	Lung	(62)
G-CSF	–	–	MDSC	Melanoma/Lung cancer/Lymphoma	–	(63)
FN	Fibroblasts	VLA-4	VEGFR1^+^ HPCs	Lung cancer	Lung	(4)
VEGF	Ovarian tumor cells	VEGFR1	MDSCs	Ovarian cancer	PN	(47)
TGF-β	Melanoma cells		Id1^high^MDSCs	Melanoma	–	(64)
SAA	ECs	TLR4	CD11b^+^ myeloid cells	Lung cancer	Lung	(65)
miRNA9	MDSCs	–	MDSCs	Lung cancer	–	(24)

### Chemokines

**Figure 1:**
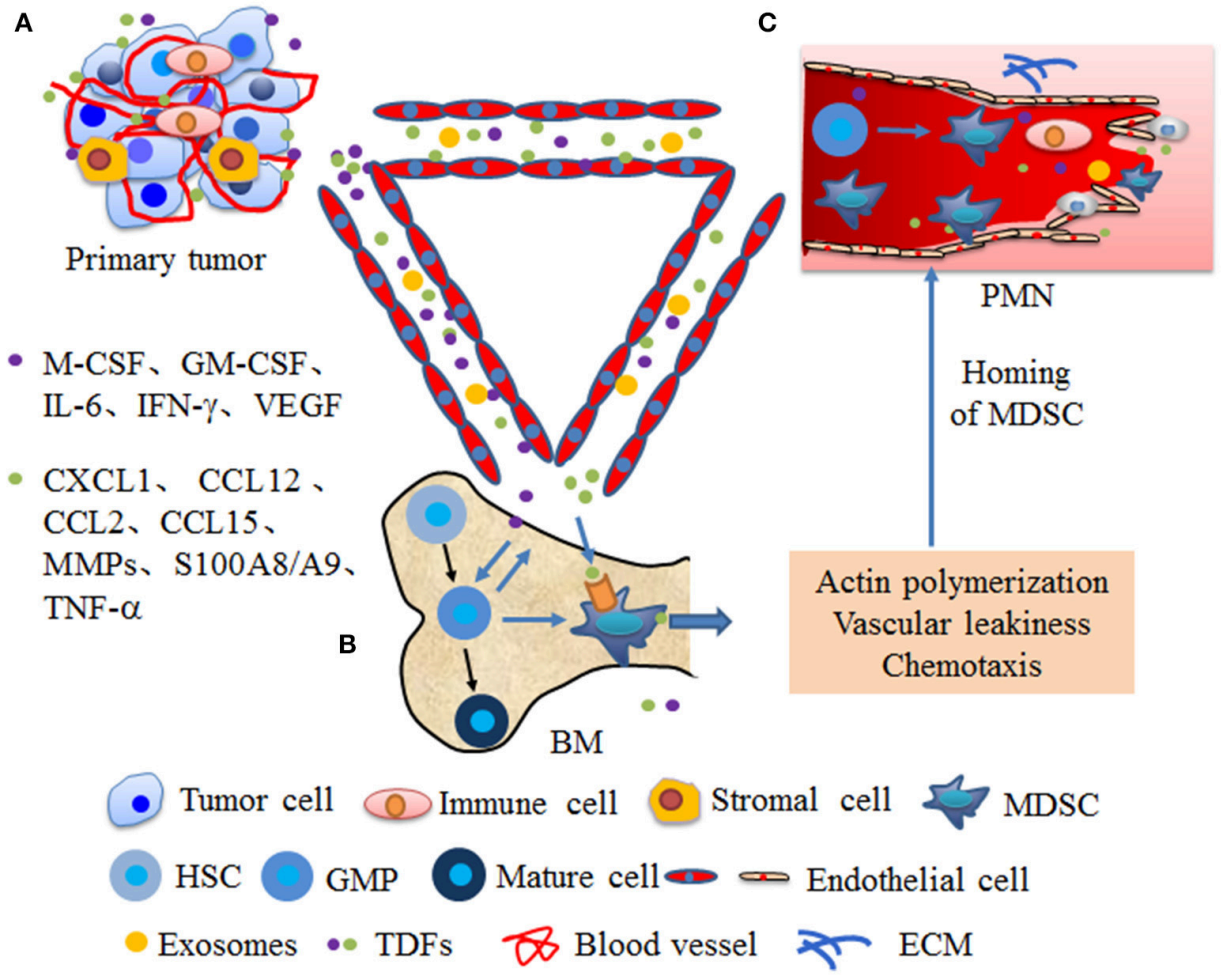
Primary tumors promote the mobilization of MDSCs from bone marrow to secondary sites. **(A)** At primary tumor site, tumor or stromal cells secrete numerous cytokines and EVs that are systemically distributed following the blood circulation. **(B)** In the bone marrow (BM), cytokines, such as macrophage-colony stimulating factor (M-CSF), granulocyte macrophage-colony stimulating factor (GM-CSF), interleukin 6 (IL-6), interferon gamma (IFN-γ), vascular endothelial growth factor (VEGF) from primary tumors promote MDSCs differentiation from granulocyte/monocyte precursor (GMP). Moreover, these cytokines mobilize MDSCs into the bloodstream through enhancing actin polymerization and vascular leakiness. **(C)** Cytokines from primary tumors, such as chemokine (C-X-C motif) ligand 1 (CXCL1), chemokine (CC motif) ligand 12 (CCL12), chemokine (CC motif) ligand 2 (CCL2), chemokine (CC motif) ligand 15 (CCL15), matrix metalloproteinases (MMPs), S100A8/A9, and tumor necrosis factor α (TNF-α) guide the homing of MDSCs to in secondary sites through chemotaxis and enhancin vascular remodeling, which create conditions conducive for MDSC mobilization to PMN. Moreover, factors or exosomes from primary tumor also enhance progenitors mobilization to the PMN and these progenitors further differentiate into MDSCs.

Chemokines and other soluble factors secreted by tumors and stromal cells are the main components that affect the migration and activation of MDSCs in the PMN. Primary tumor cells and stromal cells secreted factors and EVs drive the expansion of MDSCs within the bone marrow and enhance actin polymerization in MDSCs and vascular leakiness in the bone marrow (BM) and PMN, which create conditions conducive for the mobilization of MDSC from BM to secondary sites (Figure 1). In colorectal cancer, VEGF secretion by colorectal carcinoma cells stimulates tumor-associated macrophages (TAMs) to produce chemokine (C-X-C motif) ligand 1 (CXCL1), which recruits C-X-C motif chemokine receptor 2 (CXCR2)^+^ MDSCs to the liver tissue. The accumulated MDSCs promote PMN formation and ultimately promote liver metastases (56). CCL2 is also referred to as monocyte chemoattractant protein 1 (MCP1), was demonstrated to be a functional contributor to PMNs. In a murine liver tumor model, tumor-associated fibroblast-secreted CCL2 induces mobilization and migration of MDSCs to the PMN through chemokine receptor 2 (CCR2) (66). In a mouse breast cancer lung metastasis animal model, CCL2 also promotes MDSC migration and triggers S100A8/A9 secretion (20). In breast cancer, monocyte chemoattractant protein 1 (MCP-1) recruits PMN-MDSCs to the pre-metastatic lung and suppresses NK cell function, which promotes the formation of an immunosuppressive PMN (21). BMDCs express CCL2 to attract MDSCs via CCR2 in hedgehog-induced skin tumors (67). Furthermore, CCL12 promotes M-MDSCs to migrate to premetastatic lungs in melanoma cell-bearing mice and increases IL-1β and E-selectin expression before the arrival of tumor cells, which is beneficial for tumor cell arrest of endothelial cells (68). In addition, CCL9 is an important factor supporting MDSC recruitment to future PMNs. In colorectal cancer, CCL9 from the tumor epithelium recruits immature myeloid cells via the CCR1 receptor, which promotes tumor invasion (69). In melanoma and breast cancer-bearing mice, TGF-β regulates CCL9 production in MDSCs through p38, which shows a CCL9-CCR1 autocrine effect on MDSC survival through decreasing cell apoptosis (70). Moreover, CCL9 increases the levels of phosphorylated protein kinase B (p-PKB) and B-cell lymphoma-2 (Bcl-2) in tumor cells, which promote the survival of newly arriving tumor cells in the PMN (70). Last but not least, serum CCL15 also promotes MDSC recruitment through CCR1, which is beneficial for colorectal cancer cell metastasis to the liver (71). It is worth noting that primary tumor also enhance BM progenitors mobilization to the PMN through factors or exosomes secretion and these progenitors may further differentiate into MDSCs. Peinado et al. confirmed that melanoma exosomes reprogrammed BM progenitors toward a c-Kit^+^Tie2^+^Met^+^ pro-vasculogenic phenotype and enhanced these progenitors mobilization to the prometastatic lung through MET (7). In mouse models of metastatic lung, during the angiogenic switch, bone marrow-derived hematopoietic progenitor cells expressing VEGFR1 proliferate and mobilize to the bloodstream. These cells home to LLC cells -specific pre-metastatic lungs and form cellular clusters before the arrival of tumor cells, which metastasize to the lungs (72). Further characterization of cellular clusters revealed that these cells expressed myelomonocytic marker CD11b and secreted MMP9 (72). Overall, the role of these chemokines in the formation and evolution of PMN must be taken. Such chemokines may be the targets to block the formation of PMN.

### Integrins

Integrins are transmembrane receptors that facilitate cell-extracellular matrix adhesion. The very late antigen-4 (VLA-4) integrin is expressed by numerous cells of haematopoietic origin and possesses a key function in the cellular immune response and cancer metastasis (73). Bone marrow-derived VEGFR1^+^VLA-4^+^ HPCs migrate to the PMN and interact with resident fibroblasts through the fibronectin ligand of VLA-4, resulting in the formation of cellular clusters. These expression patterns of fibronectin and VEGFR1^+^VLA-4^+^ clusters foster a supportive microenvironment for incoming LLC or melanoma B12 cells and dictate organ-specific tumor spread (4). Blocking VLA-4 reduces tissue infiltration of M-MDSCs through inhibiting adherence to the apical side of the endothelium during the pathogenic process underlying hepatic inflammation (74). These results suggest that VLA-4 is a key molecule that regulates MDSC infiltration into tissues and may serve as an important target for blocking PMN formation. Future studies should focus on exploring strategies for blocking PMN formation based VLA-4.

### ECM Remodeling-Related Factors

The ECM remodeling-related factors contributes to many aspects of tumor progression by acting on both tumor and immune cells. In particular, ECM remodeling-related factors-mediated regulation of immunosuppression occurs through regulation of the expansion, localization, and functional activities of myeloid cells (75). The calcium binding protein S100A8/A9 is a damage-associated molecular pattern which can activate Toll-like receptor (TLR)-4 or receptor for advanced glycation end-products (RAGE). Activation of these receptors is involved in the recruitment of MDSCs. In LLC-bearing mice, S100A8 promotes MDSC recruitment through p38 and NF-κB activation in a TLR4-dependent manner (59). In mammary carcinoma cell-bearing mice, S100A8/A9 from myeloid and tumor cells bind to RAGE on MDSCs and promote MDSC migration and accumulation through the NF-κB signaling pathways (76, 77). Periostin, which is a non-structural ECM protein, is a limiting factor in the metastatic colonization of disseminated tumor cells. Periostin promotes the pulmonary accumulation of MDSCs during the early stage of breast tumor metastasis (60). In periostin-deficient MDSCs, the activation of extracellular regulated protein kinase (ERK), PKB and STAT3 and immunosuppressive functions are decreased, which accelerate breast tumor growth (60). These results indicate that periostin from MDSCs participates in PMN formation through promoting ECM remodeling and regulates the activation and function of MDSCs. In addition, periostin also elevate Lysyl oxidase (LOX) activity. LOX is an extracellular matrix, copper-dependent amine oxidase that catalyzes a key enzymatic step in the crosslinking of collagen (78). In PMN, LOX promotes the crosslinking of collagen IV in the basement membrane. Cross-linked collagen IV is essential for CD11b^+^ myeloid cell recruitment (60). CD11b^+^ cells adhere to cross-linked collagen IV and produce matrix metalloproteinase-2 (MMP2). MMP2 cleaves collagen, enhancing the invasion and recruitment of bone marrow-derived cells (62), which promote PMN formation. PMN-MDSCs are important regulators of immune responses in cancer and have been directly implicated in promotion of PMN formation. Lectin-type oxidized LDL receptor-1 (LOX-1) is a distinct surface marker for human PMN-MDSC. Endoplasmic reticulum (ER) stress converts neutrophils from healthy donors to suppressive G-MDSCs through increasing LOX-1 expression (61). In patients with hepatocellular carcinoma, ER stress promote LOX-1^+^CD15^+^ G-MDSCs expansion and suppress T cell proliferation through ROS/Arg-1(79). These results suggest that significant ER stress in a tumor-bearing host might induce PMN formation mediated by enhancement of LOX-1^+^CD15^+^ G-MDSCs -mediated suppression. In tumor-bearing mice transplanted with B16F1, Tib6, EL4, or LLC cells, tumor-secreted granulocyte colony-stimulating factor (G-CSF) mobilizes peripheral CD11b^+^Gr1^+^ cells to the pre-metastatic lung (63). VEGFA from ovarian cancer cells promotes MDSC migration and differentiation through VEGFR1, which is expressed on MDSCs, and suppress CD8^+^ T cell infiltration (48). In melanoma tumors, TGF-β mediated inhibitor of differentiation 1 (Id1) upregulation skews dendritic cell differentiation to MDSCs and mobilizes VEGFR1^+^ haematopoietic progenitor cells (HPCs) during PMN formation (64). In addition, serum amyloid A(SAA) 3, an acute phase protein, stimulates proliferative, and proinflammatory responses of keratinocytes, also participate in the formation of PMN. In LLC or B16 cell-bearing mice, serum amyloid A (SAA) 3 from endothelial cells and alveolar macrophages also attracts CD11b^+^ myeloid cells into pre-metastatic lungs (65). Therefore, extracellular matrix proteins play a major role in MDSC expansion and PMN formation. Blocking PMN formation through targeting ECM remodeling-related cytokines is an outstanding opportunity that is awaiting further research.

## Primary MDSC-Related Pro-PMN Factors and Mechanisms

**Figure 2:**
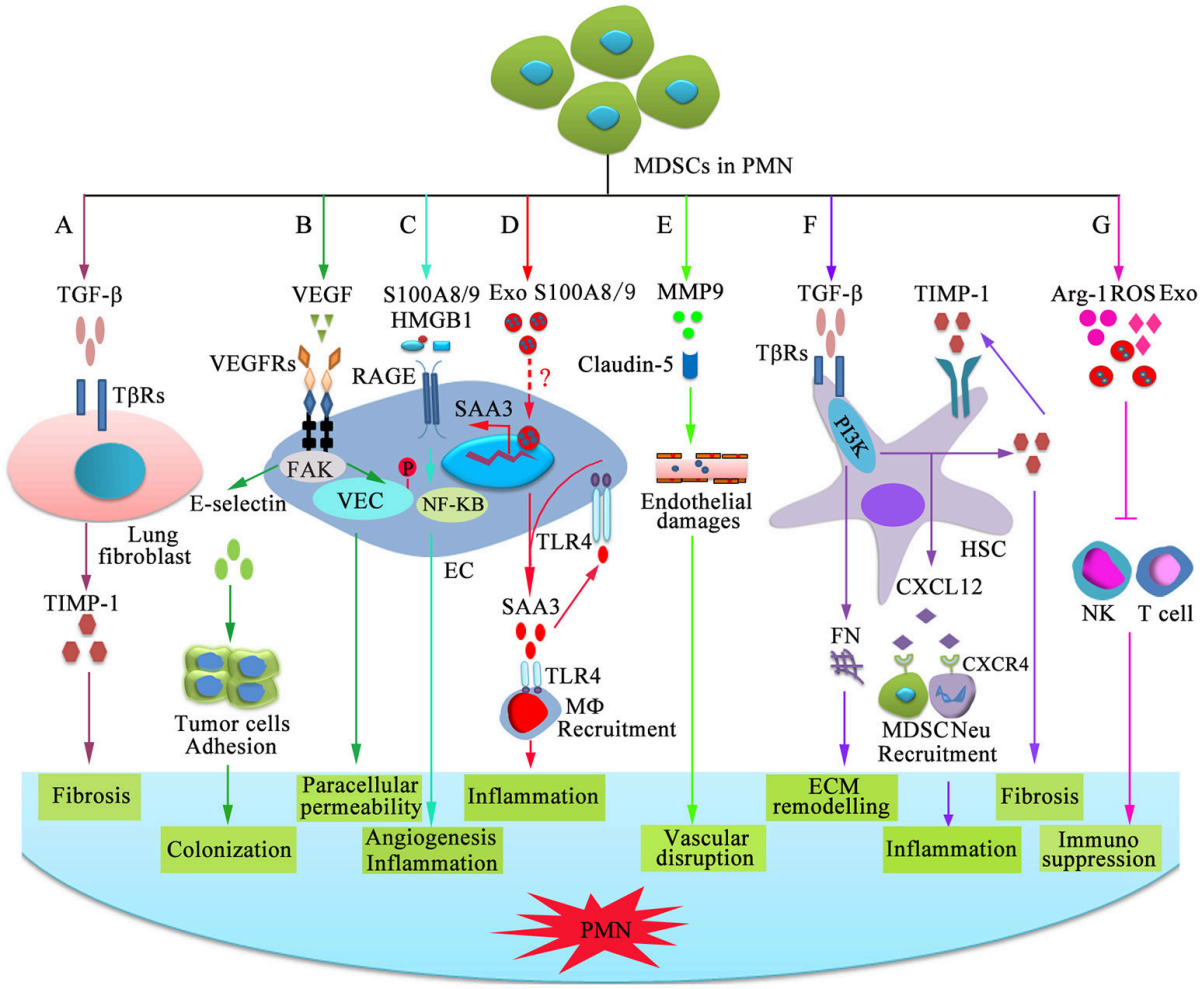
Mechanisms of MDSC-dependent promotion of PMN formation and evolution. MDSC-derived factors participate in the stepwise evolution of the PMN through regulating local resident cells, resulting in a microenvironment that encourages the settlement and outgrowth of incoming cancer cells. **(A)** MDSCs stimulate lung fibroblasts to release tissue inhibitor of metalloproteinase 1 (TIMP1) by producing TGF-β, which promotes lung fibrosis. **(B)** VEGF-dependent induction of endothelial focal adhesion kinase (FAK) promotes E-selectin upregulation, which facilitates the adhesion of circulating tumor cells. VEGF triggers FAK-dependent vascular endothelial cadherin (VEC) phosphorylation in ECs and initiates paracellular permeability. **(C)** S100A8/9 and HMGB1 bind to RAGE on ECs and promote capillary-like tube formation and production of pro-inflammatory factor through the NF-κB signaling pathway, which is beneficial for angiogenesis and inflammation. **(D)** Exosomal S100A8/9 regulates SAA3 expression by ECs. SAA3 attracts macrophages to the pre-metastatic lungs through Toll-like receptor 4 (TLR4), which is beneficial for the formation of inflammatory microenvironment. **(E)** MMP9 damages the endothelial barrier of blood vessel through damaging tight junction protein claudin-5. **(F)** TGF-β induces fibronectin (FN) production and endogenous TIMP1 expression in hepatic stellate cells (HSCs) through phosphatidylinositol 3-kinase (PI3K). FN is conducive to tissue remodeling in the liver and initiate PMN formation. Moreover, circulating TIMP1-activated HSCs express C-X-C motif chemokine 12 (CXCL12), which induces MDSC and neutrophil migration through CXCR4 and creates a microenvironment in the liver that increases its susceptibility to tumor cells. **(G)** MDSCs suppress NK-and T-cell function by secreting immunosuppressive molecules and exosomes.

Factors and cellular targets that mediate the steps of PMN formation and evolution, such as vascular leakiness, stromal education and reprogramming in organotropic sites, BMDC education and recruitment, and angiogenesis, should be validated in more detail. Dissecting PMN formation and evolution first requires examination of the earliest changes occurring within distant tissues. Current findings have identified EREG, COX2, and MMPs, which reconstitute a multi-functional vascular remodeling programme that leads to a large inflow of molecules and cells (80, 81). MDSCs are significantly increased in the lungs of mice bearing mammary adenocarcinomas before tumor cell arrival (18). The mechanisms by which MDSCs mediate PMN formation and evolution in original or distant organs remain to be elucidated, although MDSCs clearly play an immunosuppressive role through secreting Arg-1, NOS2, IL-10, COX2, ROS, TGF-β, PGE2, and IDO, sequestrating active site cysteine, decreasing L-selectin expression, and many other pathways (32). Chemokines, cytokines, growth factors and EVs from MDSCs participate in multiple stages of PMN formation and evolution. Although the individual factors of these mediators are insufficient to develop the PMN, their combined abilities result in a profound increase in the sequential steps of PMN development. In the 4T1 mammary and LLC lung carcinoma models, enhanced expression of pro-metastatic proteins in MDSCs, such as Bv8, MMP9, S100A8 and S100A9, facilitates improved PMN formation, which supports more efficient tumor cell extravasation and proliferation (44). In melanoma cell-bearing mice, the interactions of MDSCs with epithelial cells (ECs) involve an increase in vascular permeability and degradation of tight junction proteins (82). Additionally, we review the roles of MDSCs in promoting PMN formation and evolution and the possible mechanisms (Figure 2).

### TGF-β

TGF-β is a secreted polypeptide that is a key element of cancer progression toward metastasis. In colon and breast cancer mouse models, TGF-β assists in the whole metastatic dissemination process through crosstalk with cancer cells, cancer-associated fibroblasts and immune cells, which contribute to the process (83, 84). During PMN formation and evolution, MDSCs are one important source of TGF-β, which induces a series of pre-metastatic events. Exposure of pulmonary tissue to single-walled carbon nanotubes leads to TGF-β production by MDSCs, which favors the formation of a microenvironment that supports ingrowth of lung carcinoma cells (85). However, the role of TGF-β signaling in MDSC-mediated PMN formation and evolution is unclear. In the lung, CCR2^+^ M-MDSCs stimulate lung fibroblasts to release tissue inhibitor of metalloproteinase 1 (TIMP1) by producing TGF-β, which promotes lung fibrosis (86). In the liver, high TIMP1 protein levels in premalignant pancreatic lesions induce endogenous TIMP1 expression in hepatic stellate cells (HSCs) through interaction with CD63 and a process that involves the phosphatidylinositol 3-kinase (PI3K) molecule. Moreover, circulating TIMP1-activated HSCs express C-X-C motif chemokine 12 (CXCL12), which induces MDSC and neutrophil migration through CXCR4 and creates a microenvironment in the liver that increases its susceptibility to pancreatic tumor cells (57). Moreover, chronic inflammation activates human HSCs also to convert mature peripheral blood monocytes into MDSCs in a CD44-dependent fashion (87). In addition, TGF-β secretion from Kupffer cells promotes the upregulation of fibronectin production by HSCs in a pancreatic cancer mouse model, which recruit macrophages into the liver and initiate PMN formation (43). In patients with non-small cell lung cancer, TGF-β stimulates CD39 and CD73 expression on MDSCs in the PMN and inhibits T cell and NK cell activity (88). These results indicate that MDSCs may promote PMN formation through TGF-β protein secretion. Therefore, TGF-β may be an effective target for suppression of PMN formation.

### VEGF

The study of PMN formation largely focused on the lung as a target organ. In fact, the pre-metastatic lung contains many hyperpermeable vessels, activated endothelial cells, and abundant E-selectin (89, 90). The tumor microvasculature tends to be malformed, more permeable, and more tortuous than vessels in healthy tissue. These effects have been largely attributed to upregulated VEGF expression (91). In esophageal squamous cell carcinoma patients, endothelial cells within the hyperpermeable area of the PMN have been proposed to produce TGF-β in a paracrine manner, leading to fibroblast activation and VEGF release (92). Interestingly, VEGF triggers focal adhesion kinase (FAK)-dependent vascular endothelial cadherin (VEC) tyrosine (Y) 658 (VEC-Y658) phosphorylation in ECs and initiates paracellular permeability (82). Moreover, VEGFA-dependent induction of endothelial FAK or injection of recombinant VEGFA promotes E-selectin upregulation, and inhibition of endothelial cell FAK hinders lung metastasis (82, 90). E-selectin facilitates the adhesion of circulating tumor cells and lead to preferential homing of metastatic cancer cells to these foci and outgrowth. In tumor-bearing mice transplanted with B16F1, Tib6, EL4, or LLC cells, G-CSF secretion by the tumor mobilizes CD11b^+^Gr1^+^ myeloid cells to secrete VEGFA, which affects the tumor vasculature and promotes the formation of a pre-metastatic lung microenvironment (63). Overall, VEGF from MDSCs promotes PMN formation directly or indirectly and therefore may be candidate for blocking PMN.

### S100A8/9

A common denominator of inflammatory responses within the PMN is the S100 protein. The pro-inflammatory mediator S100A8/A9 is abundant at inflammatory sites. S100A8/A9 is involved in processes such as enhancement of Ca^2+^ influx, cytokine production, immune cell recruitment and inflammation (93). The exact mechanism of S100A8/S100A9 in PMN formation is unclear, although S100A8/S100A9 is crucial for intercellular crosstalk between tumor and stromal cells during PMN establishment. Extracellular S100A8/A9 from MDSCs and tumor cells stimulates macrophage polarization toward the tumor-promoting M2 phenotype, and this conversion switches off IL-12 production, which drives the development of NK cells and tumouricidal T lymphocytes (94). Moreover, S100A8/S100A9 from mammary carcinoma cells bind to RAGE on MDSCs and promote the migration and accumulation of MDSCs through the NF-κB signaling pathways (77). In addition, secretion of S100A8/S100A9 proteins by MDSCs activates endothelial cells and MDSCs, resulting in myeloid cell recruitment in the blood and secondary lymphoid organs (77). S100 proteins and high mobility group box-1 protein (HMGB1) secreted by MDSCs are ligands of RAGE. Downstream signaling pathways of RAGE are expressed in endothelial cells and MDSCs. HMGB1 effectively promotes human pulmonary microvascular endothelial cell migration and capillary-like tube formation through the ERK/P38/Src signaling pathway (95). Thus, S100A8/S100A9 may maintain an autocrine feedback loop that leads to MDSC recruitment within the PMN.

Interestingly, human breast cancer cell-derived exosomes prepare the PMN by activating Src phosphorylation and pro-inflammatory S100 gene expression in organ-specific cells (42). Moreover, MDSC exosomes induce chemotaxis of MDSCs themselves through their S100A8 and A9 content and promote M2 macrophage polarization in breast cancer model mice (96). In pancreatic cancer model mice, TGF-β signaling-induced fibronectin (FN) upregulation induces macrophage recruitment to the liver, which promotes liver PMN formation (43). Upon CCL2 stimulation, exosomal S100A8/9 produced by primary LLC or B16 cells are delivered systemically to the pre-metastatic lung endothelium and regulate SAA3 expression by endothelial cells and alveolar macrophages through stimulating the SAA3 promoter, which attracts CD11b^+^ myeloid cells to the pre-metastatic lungs (65). Furthermore, SAA3 also binds to TLR4 on lung endothelial cells and macrophages (65). Pancreatic cancer cell-derived exosomes initiate PMN formation in the liver through macrophage migration inhibitory factor (MIF) (43). This S100A8-SAA3-TLR4 cascade establishes the PMN. These results suggest that exosomal S100A8/9 play an important role in PMN formation, although the exact mechanism remains to be clarified.

### MMP9

MMPs from invasive endothelial cells or bone marrow derived-progenitor cells govern degradation of the extracellular matrix, basement membrane, and interstitial stroma, all of which are essential events during the formation of new blood vessels (91). MMP9 is a member of a family of zinc-containing endopeptidases and is maintained at high levels in the PMN (72, 97). Activation of endothelial MMP9 leads to damage of the endothelial barrier (98). Inhibiting MMP9 activity can decrease vascular permeability and improve stroke (99). Moreover, in a breast cancer mouse model, altered vascular integrity is manifested through hyperpermeability in the PMN, aberrant morphology of the vascular endothelium and breakdown of the vascular basement membrane (89). MMP9 also damaged vessel stability through sequestering vascular VEGF and TGF-β in the ECM (100). In fact, vascular permeability and neo-angiogenesis generation favor the initial extravasation and subsequent metastatic growth of tumor cells into pre-metastatic organs (101). MMP9 plays crucial roles in ECM remodeling and the angiogenic switch that supports formation of the PMN (102). MMP9 produced by MDSCs causes abnormal and leaky vasculature as well as restructuring of collagen in the basement membrane of blood vessels in the pre-metastatic lung (103). In mammary adenocarcinoma-bearing mice, CD11b^+^Gr-1^+^ myeloid progenitor cells are significantly increased in the pre-metastatic lung before tumor cell arrival and produce a large amount of MMP9, which promotes aberrant vasculature formation and leads to the formation of a proliferative, immunosuppressive and inflamed PMN in the lung (18). Moreover, ablation of MMP9 results in aberrant vasculature normalization, improvement of host immune surveillance, and diminished lung metastasis (18). Therefore, MMP9 is a crucial regulator of mobilization of bone marrow-derived endothelial cell and progenitor cell recruitment, ECM remodeling, and the angiogenic switch, which are intimately involved in regulating vascular integrity in the PMN. These results suggest that MMP9 from MDSCs or tissue-resident cells in the pre-metastatic lung destroys the vasculature stability and immune balance, resulting in PMN formation and evolution.

## Exosomes in MDSC Recruitment and PMN Formation

Exosomes (30–150 nm) are one type of membrane vesicle of endocytic origin and are secreted into the extracellular space by most cell types. Exosomes perform many biological functions, particularly intercellular communication through delivering functional proteins, mRNAs, and miRNAs into target cells following the internalization of exosomes. Cumulative evidence has suggested that tumor exosomes fuse with resident cells in the PMN and transfer their cargo, including genetic material (DNA, mRNA and miRNA), metabolites (lipids and small metabolites) and proteins, which are closely associated with the initiation, formation, and evolution of the PMN (101, 104, 105).

Exosomes shed by tumor cells have been shown to contribute to MDSC recruitment. For example, membrane-associated Hsp72 from colon carcinoma CT26, lymphoma EL4, and embryo fibroblast NIH/3T3 cells and human lung adenocarcinoma-derived exosomes mediates the STAT3-dependent immunosuppressive function of MDSCs (58). Another study show that melanoma cell-derived exosomes promote PMN formation by educating bone marrow progenitor cells toward a pro-metastatic phenotype through the MET protein (12) (Table 2). Pancreatic tumor-derived exosomes expressing MIF promote TGF-β secretion from Kupffer cells, which stimulates fibronectin secretion from hepatic stellate cells and recruits myeloid CD11b^+^ cells to the PMN in the liver (43). The pro-inflammatory proteins S100A8/S100A9 are abundant in MDSC exosomes from breast cancer model mice and is chemotactic for MDSCs *in vitro* (96). Therefore, exosomes from primary tumors play important roles in MDSC recruitment in secondary organ. The blockade of critical exosomes or their cargo is beneficial for inhibiting the accumulation and activation of MDSCs in the PMN.

Exosomes enhance the systematic entry of cancer cells along the metastatic cascade. Therefore, understanding the biology of MDSC exosomes in the PMN is important. Mass spectrometry results show that MDSC exosomes from breast cancer model mice carry biologically active components, such as metabolic enzymes, transcription factors, and proteins relevant for immunomodulation (96). MDSC exosomes also carry many surface glycoproteins and several shared ligand receptor pairs, indicating that MDSC exosomes are well equipped for binding (106). In the following paragraphs, we will further examine the possible roles of MDSC exosomes in diverse mechanisms related to PMN formation and evolution, which are favorable for inhibiting PMN establishment at secondary organs and consequent metastatic outgrowth.

The integrin on the surface of breast cancer cell exosomes promotes immature myeloid cell homing to the PMN and increases activation of S100 genes and Src signaling in the PMN in the lung and liver (7). LLC or B16/F10 cell-derived exosomal RNA activates alveolar epithelial TLR3 and consequently induces chemokine secretion in the lung and promotes neutrophil recruitment, which also promotes lung PMN formation (104). Therefore, the interactions of MDSC exosomes and cargo with ECs need to be clarified further. In cancer patients, intratumoural and peripheral MDSCs inevitably shed large exosomes, which are involved in PMN formation and evolution, although the exact mechanism needs to be further clarified. Breast cancer cell exosomal miR-210 promotes angiogenesis and metastasis by regulating EC behavior (107, 108). Interestingly, HIF-1α can induce miR-210 overexpression in MDSCs and increase arginase activity and nitric oxide production (108), although miR-210 expression in MDSC exosomes needs to be further clarified. A study showed that MDSC exosomal miR-126a promoted lung metastasis by breast tumors (38) (Table 3). Moreover, melanoma exosomal miR-9 activates the JAK-STAT pathway through reducing the SOCS5 levels in ECs, which promotes endothelial cell migration and tumor angiogenesis (126). CREB regulates miR-9 expression and inhibits MDSC differentiation by targeting runt-related transcription factor 1 (RUNX1) (24). The miR-9 expression profile in MDSC exosomes needs to be identified, and the interactions between miR-9 and ECs need to be further investigated. MDSCs express the advanced glycosylation end-product-specific receptor ligands S100A8/9, which can contribute to activation of inflammatory/immunosuppressive genes. MDSC exosomes polarize macrophages toward a tumor-promoting type 2 phenotype and possess S100A8/A9 chemotactic activity (96). G-MDSC exosomal Arg-1 inhibits T cell proliferation (127). Clearly, many cargoes within MDSC exosomes participate in function modulation and metabolic reprogramming of immune and stromal cells.

**Table 3:** Molecules associated with the blockade of MDSC expansion and recruitment.

**Molecules**	**Cancer type**	**Phenotype**	**Species**	**References**
**VITAMIN DERIVATIVES**				
1α,25-hydroxy vitamin D3	HNSCC	MDSCs	Human	(109)
ATRA	Fibrosarcomas	MDSCs	Mouse	(110)
	Mammary adenocarcinomas	MDSCs	Mouse	(110)
	Renal cell carcinoma	MDSCs	Human	(111)
Vitamin D	CLL	CD14^+^HLA-DR^low^ MDSCs	Human	(112)
**AMINO-BISPHOSPHONATE**				
ZA	Mesothelioma	MDSCs	Mouse	(113)
	Myeloma	MDSCs	Mouse	(114)
	Pancreatic cancer	CD15^+^CD11b^+^ MDSCs	Human	(115)
	Pancreatic cancer	MDSCs	Mouse	(115)
	Breast cancer	MDSCs	Mouse	(116)
**ANTIBODIES**				
Anti-VEGFR-2 Ab	Melanoma and prostate tumor	M-MDSCs	Mouse	(117)
Anti-Gr1 Ab	Lung cancer	MDSCs	Mouse	(118)
	Myeloma	MDSCs	Mouse	(119)
MD5-1 mAb	Lymphoma	MDSCs	Mouse	(120)
DS-8273a mAb	Advanced cancers	MDSCs	Human	(121)
Anti-CD33 Ab	Myelodysplastic syndrome	CD33^+^HLA-DR^−^Lin^−^MDSCs	Human	(122)
Anti-KIT mAb	Colon cancer	M-MDSCs	Mouse	(123)
Anti-ENO1 mAb	Pancreatic ductal adenocarcinoma	MDSCs	Mouse	(124)
Anti-DC-HIL mAb	Colorectal cancer	M-MDSCs	Mouse	(125)

These results indicate that MDSC exosomes are favorable for the establishment of an inflammatory and immunosuppressive microenvironment that is a supportive niche for the arrival of tumor cells. The potential impact of MDSC exosomes on regulation of the PMN is definite, although the detailed mechanism still needs further exploration.

## Potential Application of MDSCs in PMN Detection and Therapy

Clinical establishment of PMN detection technology could help patients optimize the selection of monitoring and intervention during therapy. Nevertheless, no effective clinical techniques are available to detect the PMN at present. Early detection of the PMN before radiographic evidence of the metastatic niche remains a challenge. Following immune cells or related molecules using a radiographic method provides an opportunity to identify the PMN. For instance, whole body imaging of lymphovascular niches is used to identify the premetastatic roles of melanoma in mice (128). However, the lack of specific tracking probes hinders the application of positron emission tomography (PET) and nuclear magnetic resonance (NMR) for PMN detection. Considering the crucial role of MDSCs in pre-metastatic tissue priming and the abundance of S100A8/A9 in MDSCs, initiation of the PMN can most likely be predicted by the MDSC abundance, which is reflected by MSDC surface molecules or cytokines. Researchers have developed a method that uses antibody-based single-photon emission computed tomography (SPECT) for detection of S100A8/A9 *in vivo* as an imaging marker for pre-metastatic tissue priming (20). However, because MDSCs are not the only source of S100A8/A9, more MDSC-related molecules should be tested. Published studies have proven the roles of exosome-mediated PMN formation with diverse mechanisms.

Study showed that pancreatic cancer cell-derived exosomes initiated PMN formation in the liver through MIF (43). Moreover, human breast cancer cell-derived exosomal integrins (ITGs) direct organ-specific colonization by fusing with resident target cells in a tissue-specific fashion, thereby initiating PMN formation (7). Those tumor exosomal cargoes in plasma assist with the diagnosis and prognostic assessment of the corresponding diseases. However, those tumor exosomal cargoes play a limited role in PMN detection, because there is no effective tracer for these molecules and their distribution profiles in the pre-metastatic microenvironment are unclear. MDSC exosomes package various molecules, including S100A8/9 (96), miR-126a (38), and Arg-1 (127), which are involved in PMN formation and evolution. Moreover, MDSC exosomes express CD11b molecules (106), which provide the possibility for an exosome trace. Therefore, MDSC exosomes have potential application value for detection of the PMN.

Currently, no clinical agents are a specific target therapy for the PMN, although targeted therapies directed against establishment of the PMN can potentially inhibit metastasis in mice. In the earliest PMN event, ECM remodeling and the formation of blood clots lead to the loss of vascular integrity, which causes increased vasculature permeability. In turn, the increased vasculature permeability is beneficial for the ability of macromolecules and cells to cross endothelial barriers, which leads to ECM remodeling and destruction of vascular integrity. On the other hand, vascular leakiness leads to an abnormal microenvironment that is characterized by interstitial hypertension (elevated hydrostatic pressure outside the blood vessels). Therefore, targeting drugs to the PMN is difficult due to the increased permeability of the vasculature at the PMN (19). Encouragingly, specific targeting of PMN components reduces metastasis in preclinical models. In breast cancer, inhibition of LOX activity abrogates the formation of tumor-driven focal pre-metastatic bone lesions (129). In mice, the formation of pre-metastatic cellular clusters can be abrogated by preventing VEGFR1 function using antibodies or by removing VEGFR1^+^ cells from the bone marrow (4). Blocking SAA3-TLR4 function during the pre-metastatic phase can prevent formation of the pulmonary PMN (65). Abrogation of HPC clusters within pre-metastatic organs by either a VEGFR1 antibody or depletion of VEGFR1^+^ BMDCs reduces the metastasis of LLC or B16 cells to lung tissue (4). MDSCs play a crucial role in PMN formation and evolution and present strategic therapeutic potential. The use of low doses of approved chemotherapeutic drugs, such as 5-fluorouracil, gemcitabine, and fludarabine, represents the most promising, and feasible strategy to reduce the intratumoural numbers of MDSCs (130). In addition, small molecules, such as vitamin derivatives (112), amino-bisphosphonate (113), and antibodies (117), have been found to block MDSC expansion and recruitment (Table 3). Therefore, strategies to eliminate MDSCs and their related molecules and exosomes will help prevent PMN formation.

### Vitamin Derivatives

Vitamins A and D may aid in MDSC differentiation to more mature cells through an unknown mechanism, which has been reviewed (131, 132). The efficacy of 1α, 25-hydroxyvitamin D3 was observed in mice with lung cancer and patients with non-small cell lung and squamous cell carcinoma of the head and neck (109, 133, 134). In addition, all-trans-retinoic acid (ATRA) is a derivative of vitamin A with antiproliferative properties. ATRA targets genes responsible for cell maturation that are less likely to favor tumor growth by maturing MDSCs into DCs, granulocytes, and monocytes (135). In mice with fibrosarcomas and mammary adenocarcinomas, ATRA also enhance antitumour T cell responses (110). Clinical trials have shown that renal cell carcinoma patients with high serum ATRA concentrations have fewer peripheral blood MDSCs and improved T cell responses (111). Some new discoveries have been made concerning the regulation of MDSCs by vitamin derivatives. miR-155 induces MDSC expansion via targeting SH2 domain-containing inositol 5′-phosphatase 1 (SHIP1), leading to STAT3 activation (136). In B cell-derived chronic lymphocytic leukemia (CLL), transfer of tumor cell exosomal miR-155 contributes to CLL cell-mediated MDSC induction, which can be disrupted by vitamin D (112). Last but not least, in a model of lipopolysaccharide-induced immunosuppression, ATRA decreases the generation of MDSCs by reducing CD34^+^ precursor cell proliferation (137). Therefore, vitamin derivatives may be candidates for blocking PMN and need to be thoroughly studied through clinical trials.

### Amino-Bisphosphonate

Amino-bisphosphonate has been suggested to work as an immune modulator and therefore may be applicable as an antitumour agent that can prolong disease-free survival in cancer patients. Zoledronic acid (ZA) is a potent amino-bisphosphonate that targets the mevalonate pathway in myeloid cells. Zoledronic acid was previously shown to target MDSCs. In mesothelioma, ZA suppress TAM differentiation from MDSCs, leading to a reduced level of TAM-associated cytokines in the tumor microenvironment (113). In myeloma-challenged mice, ZA inhibits the expansion of MDSCs and bone lesions (114). In pancreatic cancer mice, ZA impairs intratumoural MDSC accumulation, resulting in a delayed tumor growth rate, prolonged median survival, and increased recruitment of T cells to the tumor (115). Amino-bisphosphonates contribute to specific MMP-9 inhibitory activity (116). In mammary tumor model mice, amino-bisphosphonates significantly reduce MDSC expansion in both the bone marrow and peripheral blood by decreasing the serum pro-MMP-9 and VEGF levels (116). These studies reinforce the importance of amino-bisphosphonate t in preventing the PMN formation and evolution.

### Antibodies

Antibodies are widely used as efficient agents for eliminating MDSCs, although their efficacies for each MDSC subtype (G-MDSCs and M-MDSCs) are controversial. For example, in melanoma and prostate tumor model mice, an anti-VEGFR-2 antibody suppresses MDSC-mediated angiogenesis through MMP-9 inhibition (117). Moreover, an anti-Gr1 antibody (RB6-8C5) is widely used as an efficient agent to eliminate MDSCs in mice. Zhang et al. (118) found that an anti-Gr1 antibody reduced MDSCs by one-third in the tumors of 3LL cell-bearing mice. Vincent Hurez used an anti-Gr1 monoclonal antibody that reduced MDSCs by 50–75% in the spleens of B16-bearing mice (119). In addition, MDSCs are sensitive to TNF-related apoptosis–induced ligand receptor 2 (TRAIL-R2) agonists. DR5, which is a TRAIL-R, plays an important role in MDSC survival. The MD5-1 mAb, which is an agonistic DR5 antibody, dramatically improves immune responses in tumor-bearing mice. In mice bearing large EL4 tumors, treatment with the MD5-1 mAb strongly decreases the accumulation of both MDSC subsets in the tumor, and this effect is quite specific for MDSCs without affecting DCs and macrophages (120). In 16 patients with advanced cancers, the agonistic TRAIL-R2 antibody DS-8273a selectively targeted MDSCs and resulted in reduction of the elevated numbers of MDSCs in the peripheral blood of most patients (121). In myelodysplastic syndrome, BI 836858, which is a Fc-engineered monoclonal antibody against CD33, also reduce MDSCs by antibody-dependent cellular cytotoxicity and block CD33 downstream signaling, thereby preventing immunosuppressive cytokine secretion (122). In colon 26 cell-bearing mice, the anti-KIT IgG1 mAb KTN0158 promoted immune responses by selectively reducing immunosuppressive M-MDSCs (123). In pancreatic ductal adenocarcinoma-bearing mice, a mAb targeting pancreatic ductal adenocarcinoma-associated antigen α-enolase (ENO1) inhibited *in vivo* infiltration of MDSCs into the tumor microenvironment and attenuated their restraint of the effector T cell response (124). Last but not least, in colorectal cancer with high blood DC-HIL^+^ MDSC levels, an anti-DC-HIL mAb attenuated tumor progression by reducing MDSCs in the tumor microenvironment (125). These works provide a foundation for the development of a novel group of therapies for the PMN aimed at MDSCs. The combined use of these antibodies may more effectively prevent the formation and evolution of PMN through targeting MDSCs.

## Conclusions and Perspectives

Taken together, the presented findings show that MDSC-derived TGF-β, S100A8/A9, VEGF, and exosomes promote PMN formation and metastasis through crosslinking with the immune system, fibroblasts, endothelial cells, and hepatic stellate cells. The main processes and mechanisms involve the induction of vascular leakiness, ECM remodeling, immunosuppression, and inflammation, although the exact mechanism remains to be confirmed. Because MDSCs play pivotal roles in PMN formation and evolution, developing strategies based on MDSCs for detection of the PMN at its earliest stages is realistic. Understanding the cross-talk between MDSCs and resident cells in pre-metastatic organs is essential for PMN targeting. Most of the work exploring PMN formation relies on mouse models of metastasis, and our understanding of PMN biology is mostly based on studies of lung or liver metastases. Some obstacles remain for clarifying the clinical traits of PMN and obtaining premetastatic tissues from patients. More clinical research is needed, and better imaging techniques for PMN detection should be developed.

## Availability of Data and Material

The material supporting the conclusion of this review has been included within the article.

## Author Contributions

YW and SW designed the study. YW and NG drafted the manuscript. YD prepared the tables and figures. All authors read and approved the final manuscript.

### Conflict of Interest Statement

The authors declare that the research was conducted in the absence of any commercial or financial relationships that could be construed as a potential conflict of interest.

## References

[1] Paget S. The distribution of secondary growths in cancer of the breast. Cancer Metastasis Rev. (1989) 8:98–101. 2673568

[2] Gupta GP Massague J. Cancer metastasis: building a framework. Cell (2006) 127:679–95. 10.1016/j.cell.2006.11.001 17110329

[3] Poste G Fidler IJ. The pathogenesis of cancer metastasis. Nature (1980) 283:139–46. 6985715 10.1038/283139a0

[4] Kaplan RN Riba RD Zacharoulis S Bramley AH Vincent L Costa C. VEGFR1-positive haematopoietic bone marrow progenitors initiate the pre-metastatic niche. Nature (2005) 438:820–7. 10.1038/nature04186 16341007 PMC2945882

[5] Hiratsuka S Nakamura K Iwai S Murakami M Itoh T Kijima H. MMP9 induction by vascular endothelial growth factor receptor-1 is involved in lung-specific metastasis. Cancer Cell (2002) 2:289–300. 10.1016/S1535-6108(02)00153-8 12398893

[6] Tichet M Prod'Homme V Fenouille N Ambrosetti D Mallavialle A Cerezo M. Tumour-derived SPARC drives vascular permeability and extravasation through endothelial VCAM1 signalling to promote metastasis. Nat Commun. (2015) 6:6993. 10.1038/ncomms7993 25925867

[7] Hoshino A Costa-Silva B Shen TL Rodrigues G Hashimoto A Tesic Mark M. Tumour exosome integrins determine organotropic metastasis. Nature (2015) 527:329–35. 10.1038/nature15756 26524530 PMC4788391

[8] Yang WW Yang LQ Zhao F Chen CW Xu LH Fu J. Epiregulin promotes lung metastasis of salivary adenoid cystic carcinoma. Theranostics (2017) 7:3700–14. 10.7150/thno.19712 29109770 PMC5667342

[9] Zhang C Zhou C Wu XJ Yang M Yang ZH Xiong HZ. Human CD133-positive hematopoietic progenitor cells initiate growth and metastasis of colorectal cancer cells. Carcinogenesis (2014) 35:2771–7. 10.1093/carcin/bgu192 25269803

[10] Zhang Y Davis C Ryan J Janney C Pena MM. Development and characterization of a reliable mouse model of colorectal cancer metastasis to the liver. Clin Exp Metastasis (2013) 30:903–18. 10.1007/s10585-013-9591-8 23748471 PMC3836876

[11] Wong CC Gilkes DM Zhang H Chen J Wei H Chaturvedi P. Hypoxia-inducible factor 1 is a master regulator of breast cancer metastatic niche formation. Proc Natl Acad Sci USA. (2011) 108:16369–74. 10.1073/pnas.1113483108 21911388 PMC3182724

[12] Peinado H Aleckovic M Lavotshkin S Matei I Costa-Silva B Moreno-Bueno G. Melanoma exosomes educate bone marrow progenitor cells toward a pro-metastatic phenotype through MET. Nat Med. (2012) 18:883–91. 10.1038/nm.2753 22635005 PMC3645291

[13] Seubert B Grunwald B Kobuch J Cui H Schelter F Schaten S. Tissue inhibitor of metalloproteinases (TIMP)-1 creates a premetastatic niche in the liver through SDF-1/CXCR4-dependent neutrophil recruitment in mice. Hepatology (2015) 61:238–48. 10.1002/hep.27378 25131778 PMC4280301

[14] Kowanetz M Wu X Lee J Tan M Hagenbeek T Qu X. Granulocyte-colony stimulating factor promotes lung metastasis through mobilization of Ly6G+Ly6C+ granulocytes. Proc Natl Acad Sci USA. (2010) 107:21248–55. 10.1073/pnas.1015855107 21081700 PMC3003076

[15] Casbon AJ Reynaud D Park C Khuc E Gan DD Schepers K. Invasive breast cancer reprograms early myeloid differentiation in the bone marrow to generate immunosuppressive neutrophils. Proc Natl Acad Sci USA. (2015) 112:E566–75. 10.1073/pnas.1424927112 25624500 PMC4330753

[16] Granot Z Henke E Comen EA King TA Norton L Benezra R. Tumor entrained neutrophils inhibit seeding in the premetastatic lung. Cancer Cell (2011) 20:300–14. 10.1016/j.ccr.2011.08.012 21907922 PMC3172582

[17] Lavender N Yang J Chen SC Sai J Johnson CA Owens P. The Yin/Yan of CCL2: a minor role in neutrophil anti-tumor activity *in vitro* but a major role on the outgrowth of metastatic breast cancer lesions in the lung *in vivo*. BMC Cancer (2017) 17:88. 10.1186/s12885-017-3074-2 28143493 PMC5286656

[18] Yan HH Pickup M Pang Y Gorska AE Li Z Chytil A. Gr-1+CD11b+ myeloid cells tip the balance of immune protection to tumor promotion in the premetastatic lung. Cancer Res. (2010) 70:6139–49. 10.1158/0008-5472.can-10-0706 20631080 PMC4675145

[19] Peinado H Zhang H Matei IR Costa-Silva B Hoshino A Rodrigues G. Pre-metastatic niches: organ-specific homes for metastases. Nat Rev Cancer (2017) 17:302–17. 10.1038/nrc.2017.6 28303905

[20] Eisenblaetter M Flores-Borja F Lee JJ Wefers C Smith H Hueting R. Visualization of tumor-immune interaction - target-specific imaging of S100A8/A9 reveals pre-metastatic niche establishment. Theranostics (2017) 7:2392–401. 10.7150/thno.17138 28744322 PMC5525744

[21] Sceneay J Parker BS Smyth MJ Moller A. Hypoxia-driven immunosuppression contributes to the pre-metastatic niche. Oncoimmunology (2013) 2:e22355. 10.4161/onci.22355 23482904 PMC3583916

[22] Giles AJ Reid CM Evans JD Murgai M Vicioso Y Highfill SL. Activation of hematopoietic stem/progenitor cells promotes immunosuppression within the pre-metastatic niche. Cancer Res. (2016) 76:1335–47. 10.1158/0008-5472.can-15-0204 26719537 PMC4794356

[23] Owyong M Efe G Owyong M Abbasi AJ Sitarama V Plaks V. Overcoming barriers of age to enhance efficacy of cancer immunotherapy: the clout of the extracellular matrix. Front Cell Dev Biol. (2018) 6:19. 10.3389/fcell.2018.00019 29546043 PMC5837988

[24] Tian J Rui K Tang X Ma J Wang Y Tian X. MicroRNA-9 Regulates the differentiation and function of myeloid-derived suppressor cells via targeting Runx1. J Immunol. (2015) 195:1301–11. 10.4049/jimmunol.1500209 26091714

[25] Tian J Rui K Hong Y Wang X Xiao F Lin X Increased GITRL impairs the function of myeloid-derived suppressor cells and exacerbates primary Sjögren's syndrome. J Immunol. (2019). 10.4049/jimmunol.1801051. [Epub ahead of print]. 30760623

[26] Zhao Y Wu T Shao S Shi B Zhao Y. Phenotype, development, and biological function of myeloid-derived suppressor cells. Oncoimmunology (2016) 5:e1004983. 10.1080/2162402x.2015.1004983 27057424 PMC4801459

[27] Zhou Q Tang X Tian X Tian J Zhang Y Ma JLncRNA MALAT1 negatively regulates MDSCs in patients with lung cancer. J Cancer (2018) 14:2436–42. 10.7150/jca.24796 PMC6036894 30026840

[28] Kwak HJ Liu P Bajrami B Xu Y Park SY Nombela-Arrieta C. Myeloid cell-derived reactive oxygen species externally regulate the proliferation of myeloid progenitors in emergency granulopoiesis. Immunity (2015) 42:159–71. 10.1016/j.immuni.2014.12.017 25579427 PMC4303526

[29] Shojaei F Wu X Zhong C Yu L Liang XH Yao J. Bv8 regulates myeloid-cell-dependent tumour angiogenesis. Nature (2007) 450: 825–31. 10.1038/nature06348 18064003

[30] Maenhout SK Thielemans K Aerts JL. Location, location, location: functional and phenotypic heterogeneity between tumor-infiltrating and non-infiltrating myeloid-derived suppressor cells. Oncoimmunology (2014) 3:e956579. 10.4161/21624011.2014.956579 25941577 PMC4292540

[31] Safarzadeh E Orangi M Mohammadi H Babaie F Baradaran B. Myeloid-derived suppressor cells: Important contributors to tumor progression and metastasis. J Cell Physiol. (2018) 233:3024–36. 10.1002/jcp.26075 28661031

[32] Wang Y Tian J Wang S. The potential therapeutic role of myeloid-derived suppressor cells in autoimmune arthritis. Semin Arthritis Rheum. (2016) 45:490–5. 10.1016/j.semarthrit.2015.07.003 26272193

[33] Bronte V Brandau S Chen SH Colombo MP Frey AB Greten TF. Recommendations for myeloid-derived suppressor cell nomenclature and characterization standards. Nat Commun. (2016) 7:12150. 10.1038/ncomms12150 27381735 PMC4935811

[34] Solito S Pinton L Mandruzzato S. In Brief: Myeloid-derived suppressor cells in cancer. J Pathol. (2017) 242:7–9. 10.1002/path.4876 28097660 PMC5413806

[35] Shojaei F Zhong C Wu X Yu L Ferrara N. Role of myeloid cells in tumor angiogenesis and growth. Trends Cell Biol. (2008) 18:372–8. 10.1016/j.tcb.2008.06.003 18614368

[36] Cui TX Kryczek I Zhao L Zhao E Kuick R Roh MH. Myeloid-derived suppressor cells enhance stemness of cancer cells by inducing microRNA101 and suppressing the corepressor CtBP2. Immunity (2013) 39:611–21. 10.1016/j.immuni.2013.08.025 24012420 PMC3831370

[37] Toh B Wang X Keeble J Sim WJ Khoo K Wong WC. Mesenchymal transition and dissemination of cancer cells is driven by myeloid-derived suppressor cells infiltrating the primary tumor. PLoS Biol. (2011) 9:e1001162. 10.1371/journal.pbio.1001162 21980263 PMC3181226

[38] Deng Z Rong Y Teng Y Zhuang X Samykutty A Mu J. Exosomes miR-126a released from MDSC induced by DOX treatment promotes lung metastasis. Oncogene (2017) 36:639–51. 10.1038/onc.2016.229 27345402 PMC5419051

[39] Kumar V Patel S Tcyganov E Gabrilovich DI. The nature of Myeloid-derived suppressor cells in the tumor microenvironment. Trends Immunol. (2016) 37:208–20. 10.1016/j.it.2016.01.004 26858199 PMC4775398

[40] Tian X Ma J Wang T Tian J Zhang Y Mao L. Long non-coding RNA HOXA transcript antisense RNA Myeloid-specific 1-HOXA1 axis downregulates the immunosuppressive activity of Myeloid-derived suppressor cells in lung cancer. Front Immunol. (2018) 9:473. 10.3389/fimmu.2018.00473 29662483 PMC5890118

[41] Haverkamp JM Smith AM Weinlich R Dillon CP Qualls JE Neale G. Myeloid-derived suppressor activity is mediated by monocytic lineages maintained by continuous inhibition of extrinsic and intrinsic death pathways. Immunity (2014) 41:947–59. 10.1016/j.immuni.2014.10.020 25500368 PMC4272664

[42] Aguado BA Caffe JR Nanavati D Rao SS Bushnell GG Azarin SM. Extracellular matrix mediators of metastatic cell colonization characterized using scaffold mimics of the pre-metastatic niche. Acta Biomater. (2016) 33:13–24. 10.1016/j.actbio.2016.01.043 26844426 PMC4777643

[43] Costa-Silva B Aiello NM Ocean AJ Singh S Zhang H Thakur BK. Pancreatic cancer exosomes initiate pre-metastatic niche formation in the liver. Nat Cell Biol. (2015) 17:816–26. 10.1038/ncb3169 25985394 PMC5769922

[44] Wu CF Andzinski L Kasnitz N Kroger A Klawonn F Lienenklaus S. The lack of type I interferon induces neutrophil-mediated pre-metastatic niche formation in the mouse lung. Int J Cancer (2015) 137:837–47. 10.1002/ijc.29444 25604426

[45] Murgai M Ju W Eason M Kline J Beury DW Kaczanowska S. KLF4-dependent perivascular cell plasticity mediates pre-metastatic niche formation and metastasis. Nat Med. (2017) 23:1176–90. 10.1038/nm.4400 28920957 PMC5724390

[46] Casacuberta-Serra S Pares M Golbano A Coves E Espejo C Barquinero J. Myeloid-derived suppressor cells can be efficiently generated from human hematopoietic progenitors and peripheral blood monocytes. Immunol Cell Biol. (2017) 95:538–48. 10.1038/icb.2017.4 28108746

[47] Alfaro C Teijeira A Onate C Perez G Sanmamed MF Andueza MP. Tumor-produced interleukin-8 attracts human myeloid-derived suppressor cells and elicits extrusion of neutrophil extracellular traps (NETs). Clin Cancer Res. (2016) 22:3924–36. 10.1158/1078-0432.ccr-15-2463 26957562

[48] Horikawa N Abiko K Matsumura N Hamanishi J Baba T Yamaguchi K. Expression of vascular endothelial growth factor in ovarian cancer inhibits tumor immunity through the accumulation of myeloid-derived suppressor cells. Clin Cancer Res. (2017) 23:587–99. 10.1158/1078-0432.ccr-16-0387 27401249

[49] Hart KM Usherwood EJ Berwin BL. CX3CR1 delineates temporally and functionally distinct subsets of myeloid-derived suppressor cells in a mouse model of ovarian cancer. Immunol Cell Biol. (2014) 92:499–508. 10.1038/icb.2014.13 24613975 PMC4211619

[50] Prima V Kaliberova LN Kaliberov S Curiel DT Kusmartsev S. COX2/mPGES1/PGE2 pathway regulates PD-L1 expression in tumor-associated macrophages and myeloid-derived suppressor cells. Proc Natl Acad Sci USA. (2017) 114:1117–22. 10.1073/pnas.1612920114 28096371 PMC5293015

[51] Ridder K Sevko A Heide J Dams M Rupp AK Macas J. Extracellular vesicle-mediated transfer of functional RNA in the tumor microenvironment. Oncoimmunology (2015) 4:e1008371. 10.1080/2162402x.2015.1008371 26155418 PMC4485784

[52] Hsieh CC Chou HS Yang HR Lin F Bhatt S Qin J. The role of complement component 3 (C3) in differentiation of myeloid-derived suppressor cells. Blood (2013) 121:1760–8. 10.1182/blood-2012-06-440214 23299310 PMC3591797

[53] Wong JL Obermajer N Odunsi K Edwards RP Kalinski P. Synergistic COX2 Induction by IFNgamma and TNFalpha self-limits type-1 immunity in the human tumor microenvironment. Cancer Immunol Res. (2016) 4:303–11. 10.1158/2326-6066.cir-15-0157 26817996 PMC4877699

[54] Hossain DM Pal SK Moreira D Duttagupta P Zhang Q Won H. TLR9-targeted STAT3 silencing abrogates immunosuppressive activity of myeloid-derived suppressor cells from prostate cancer patients. Clin Cancer Res. (2015) 21:3771–82. 10.1158/1078-0432.ccr-14-3145 25967142 PMC4537814

[55] Talmadge JE Gabrilovich DI. History of myeloid-derived suppressor cells. Nat Rev Cancer (2013) 13:739–52. 10.1038/nrc3581 24060865 PMC4358792

[56] Wang D Sun H Wei J Cen B DuBois RN. CXCL1 is critical for premetastatic niche formation and metastasis in colorectal cancer. Cancer Res. (2017) 77:3655–65. 10.1158/0008-5472.can-16-3199 28455419 PMC5877403

[57] Grunwald B Harant V Schaten S Fruhschutz M Spallek R Hochst B. Pancreatic premalignant lesions secrete tissue inhibitor of metalloproteinases-1, which activates hepatic stellate cells via CD63 signaling to create a premetastatic niche in the liver. Gastroenterology (2016) 151:1011–24 e7. 10.1053/j.gastro.2016.07.043 27506299

[58] Chalmin F Ladoire S Mignot G Vincent J Bruchard M Remy-Martin JP. Membrane-associated Hsp72 from tumor-derived exosomes mediates STAT3-dependent immunosuppressive function of mouse and human myeloid-derived suppressor cells. J Clin Invest. (2010) 120:457–71. 10.1172/jci40483 20093776 PMC2810085

[59] Deguchi A Tomita T Ohto U Takemura K Kitao A Akashi-Takamura S. Eritoran inhibits S100A8-mediated TLR4/MD-2 activation and tumor growth by changing the immune microenvironment. Oncogene (2016) 35:1445–56. 10.1038/onc.2015.211 26165843

[60] Wang Z Xiong S Mao Y Chen M Ma X Zhou X. Periostin promotes immunosuppressive premetastatic niche formation to facilitate breast tumour metastasis. J Pathol. (2016) 239:484–95. 10.1002/path.4747 27193093

[61] Condamine T Dominguez GA Youn JI Kossenkov AV Mony S Alicea-Torres K. Lectin-type oxidized LDL receptor-1 distinguishes population of human polymorphonuclear myeloid-derived suppressor cells in cancer patients. Sci Immunol. (2016) 1:aaf8943. 10.1126/sciimmunol.aaf8943 28417112 PMC5391495

[62] Erler JT Bennewith KL Cox TR Lang G Bird D Koong A. Hypoxia-induced lysyl oxidase is a critical mediator of bone marrow cell recruitment to form the premetastatic niche. Cancer Cell (2009) 15:35–44. 10.1016/j.ccr.2008.11.012 19111879 PMC3050620

[63] Shojaei F Wu X Qu X Kowanetz M Yu L Tan M. G-CSF-initiated myeloid cell mobilization and angiogenesis mediate tumor refractoriness to anti-VEGF therapy in mouse models. Proc Natl Acad Sci USA. (2009) 106:6742–7. 10.1073/pnas.0902280106 19346489 PMC2665197

[64] Papaspyridonos M Matei I Huang Y do Rosario Andre M Brazier-Mitouart H Waite JC. Id1 suppresses anti-tumour immune responses and promotes tumour progression by impairing myeloid cell maturation. Nat Commun. (2015) 6:6840. 10.1038/ncomms7840 25924227 PMC4423225

[65] Hiratsuka S Watanabe A Sakurai Y Akashi-Takamura S Ishibashi S Miyake K. The S100A8-serum amyloid A3-TLR4 paracrine cascade establishes a pre-metastatic phase. Nat Cell Biol. (2008) 10:1349–55. 10.1038/ncb1794. 18820689

[66] Yang X Lin Y Shi Y Li B Liu W Yin W. FAP promotes immunosuppression by cancer-associated fibroblasts in the tumor microenvironment via STAT3-CCL2 signaling. Cancer Res. (2016) 76:4124–35. 10.1158/0008-5472.can-15-2973 27216177

[67] Fan Q Gu D Liu H Yang L Zhang X Yoder MC. Defective TGF-beta signaling in bone marrow-derived cells prevents hedgehog-induced skin tumors. Cancer Res. (2014) 74:471–83. 10.1158/0008-5472.can-13-2134-t 24282281 PMC3963525

[68] Shi H Zhang J Han X Li H Xie M Sun Y. Recruited monocytic myeloid-derived suppressor cells promote the arrest of tumor cells in the premetastatic niche through an IL-1beta-mediated increase in E-selectin expression. Int J Cancer (2017) 140:1370–83. 10.1002/ijc.30538 27885671

[69] Kitamura T Fujishita T Loetscher P Revesz L Hashida H Kizaka-Kondoh S. Inactivation of chemokine (C-C motif) receptor 1 (CCR1) suppresses colon cancer liver metastasis by blocking accumulation of immature myeloid cells in a mouse model. Proc Natl Acad Sci USA. (2010) 107:13063–8. 10.1073/pnas.1002372107 20616008 PMC2919974

[70] Yan HH Jiang J Pang Y Achyut BR Lizardo M Liang X. CCL9 induced by TGFbeta signaling in myeloid cells enhances tumor cell survival in the premetastatic organ. Cancer Res. (2015) 75:5283–98. 10.1158/0008-5472.can-15-2282-t 26483204 PMC5120555

[71] Inamoto S Itatani Y Yamamoto T Minamiguchi S Hirai H Iwamoto M. Loss of SMAD4 promotes colorectal cancer progression by accumulation of myeloid-derived suppressor cells through the CCL15-CCR1 chemokine axis. Clin Cancer Res. (2016) 22:492–501. 10.1158/1078-0432.ccr-15-0726 26341919

[72] Kaplan RN Psaila B Lyden D. Bone marrow cells in the 'pre-metastatic niche': within bone and beyond. Cancer Metastasis Rev. (2006) 25:521–9. 10.1007/s10555-006-9036-9 17186383

[73] Schlesinger M Bendas G. Contribution of very late antigen-4 (VLA-4) integrin to cancer progression and metastasis. Cancer Metastasis Rev. (2015) 34:575–91. 10.1007/s10555-014-9545-x 25564456

[74] Resheq YJ Menzner AK Bosch J Tickle J Li KK Wilhelm A. Impaired transmigration of myeloid-derived suppressor cells across human sinusoidal endothelium is associated with decreased expression of CD13. J Immunol. (2017) 199:1672–81. 10.4049/jimmunol.1600466 28739875

[75] Sangaletti S Chiodoni C Tripodo C Colombo MP. Common extracellular matrix regulation of myeloid cell activity in the bone marrow and tumor microenvironments. Cancer Immunol Immunother. (2017) 66:1059–67. 10.1007/s00262-017-2014-y 28501940 PMC11029001

[76] Cheng P Corzo CA Luetteke N Yu B Nagaraj S Bui MM. Inhibition of dendritic cell differentiation and accumulation of myeloid-derived suppressor cells in cancer is regulated by S100A9 protein. J Exp Med. (2008) 205:2235–49. 10.1084/jem.20080132 18809714 PMC2556797

[77] Sinha P Okoro C Foell D Freeze HH Ostrand-Rosenberg S Srikrishna G. Proinflammatory S100 proteins regulate the accumulation of myeloid-derived suppressor cells. J Immunol. (2008) 181:4666–75. 10.4049/jimmunol.181.7.4666 18802069 PMC2810501

[78] Maruhashi T Kii I Saito M Kudo A. Interaction between periostin and BMP-1 promotes proteolytic activation of lysyl oxidase. J Biol Chem. (2010) 285:13294–303. 10.1074/jbc.M109.088864 20181949 PMC2857065

[79] Nan J Xing YF Hu B Tang JX Dong HM He YM. Endoplasmic reticulum stress induced LOX-1(+) CD15(+) polymorphonuclear myeloid-derived suppressor cells in hepatocellular carcinoma. Immunology (2018) 154:144–55. 10.1111/imm.12876 29211299 PMC5904716

[80] Gupta GP Nguyen DX Chiang AC Bos PD Kim JY Nadal C. Mediators of vascular remodelling co-opted for sequential steps in lung metastasis. Nature (2007) 446:765–70. 10.1038/nature05760 17429393

[81] Huang X Jia L Qian Z Jia Y Chen X Xu X. Diversity in human placental microvascular endothelial cells and macrovascular endothelial cells. Cytokine (2018) 111:287–94. 10.1016/j.cyto.2018.09.009 30269024

[82] Jean C Chen XL Nam JO Tancioni I Uryu S Lawson C. Inhibition of endothelial FAK activity prevents tumor metastasis by enhancing barrier function. J Cell Biol. (2014) 204:247–63. 10.1083/jcb.201307067 24446483 PMC3897185

[83] Tauriello DVF Palomo-Ponce S Stork D Berenguer-Llergo A Badia-Ramentol J Iglesias M. TGFbeta drives immune evasion in genetically reconstituted colon cancer metastasis. Nature (2018) 554:538–43. 10.1038/nature25492 29443964

[84] Bellomo C Caja L Moustakas A. Transforming growth factor beta as regulator of cancer stemness and metastasis. Br J Cancer (2016) 115:761–9. 10.1038/bjc.2016.255 27537386 PMC5046208

[85] Shvedova AA Kisin ER Yanamala N Tkach AV Gutkin DW Star A. MDSC and TGFbeta are required for facilitation of tumor growth in the lungs of mice exposed to carbon nanotubes. Cancer Res. (2015) 75:1615–23. 10.1158/0008-5472.can-14-2376 25744719 PMC4401633

[86] Lebrun A Lo Re S Chantry M Izquierdo Carerra X Uwambayinema F Ricci D. CCR2(+) monocytic myeloid-derived suppressor cells (M-MDSCs) inhibit collagen degradation and promote lung fibrosis by producing transforming growth factor-beta1. J Pathol. (2017) 243:320–30. 10.1002/path.4956 28799208

[87] Hochst B Schildberg FA Sauerborn P Gabel YA Gevensleben H Goltz D. Activated human hepatic stellate cells induce myeloid derived suppressor cells from peripheral blood monocytes in a CD44-dependent fashion. J Hepatol. (2013) 59:528–35. 10.1016/j.jhep.2013.04.033 23665041

[88] Li J Wang L Chen X Li L Li Y Ping Y. CD39/CD73 upregulation on myeloid-derived suppressor cells via TGF-beta-mTOR-HIF-1 signaling in patients with non-small cell lung cancer. Oncoimmunology (2017) 6:e1320011. 10.1080/2162402x.2017.1320011 28680754 PMC5486179

[89] Huang Y Song N Ding Y Yuan S Li X Cai H. Pulmonary vascular destabilization in the premetastatic phase facilitates lung metastasis. Cancer Res. (2009) 69:7529–37. 10.1158/0008-5472.can-08-4382 19773447

[90] Hiratsuka S Goel S Kamoun WS Maru Y Fukumura D Duda DG. Endothelial focal adhesion kinase mediates cancer cell homing to discrete regions of the lungs via E-selectin up-regulation. Proc Natl Acad Sci USA. (2011) 108:3725–30. 10.1073/pnas.1100446108 21321210 PMC3048115

[91] Bordeleau F Mason BN Lollis EM Mazzola M Zanotelli MR Somasegar S. Matrix stiffening promotes a tumor vasculature phenotype. Proc Natl Acad Sci USA. (2017) 114:492–97. 10.1073/pnas.1613855114 28034921 PMC5255592

[92] Noma K Smalley KS Lioni M Naomoto Y Tanaka N El-Deiry W. The essential role of fibroblasts in esophageal squamous cell carcinoma-induced angiogenesis. Gastroenterology (2008) 134:1981–93. 10.1053/j.gastro.2008.02.061 18439605 PMC2562524

[93] Donato R Cannon BR Sorci G Riuzzi F Hsu K Weber DJ. Functions of S100 proteins. Curr Mol Med. (2013) 13:24–57. 10.2174/1566524011307010024 22834835 PMC3707951

[94] Ostrand-Rosenberg S. Myeloid-derived suppressor cells: more mechanisms for inhibiting antitumor immunity. Cancer Immunol Immunother. (2010) 59:1593–600. 10.1007/s00262-010-0855-8 20414655 PMC3706261

[95] Liu Z Wang J Xing W Peng Y Quan J Fan X. LPS binding to HMGB1 promotes angiogenic behavior of endothelial cells through inhibition of p120 and CD31 via ERK/P38/Src signaling. Eur J Cell Biol. (2017) 96:695–704. 10.1016/j.ejcb.2017.07.004 28818340

[96] Burke M Choksawangkarn W Edwards N Ostrand-Rosenberg S Fenselau C. Exosomes from myeloid-derived suppressor cells carry biologically active proteins. J Proteome Res. (2014) 13:836–43. 10.1021/pr400879c 24295599 PMC3946337

[97] Yao H Price TT Cantelli G Ngo B Warner MJ Olivere L. Leukaemia hijacks a neural mechanism to invade the central nervous system. Nature (2018) 560:55–60. 10.1038/s41586-018-0342-5 30022166 PMC10257142

[98] Spampinato SF Merlo S Sano Y Kanda T Sortino MA. Astrocytes contribute to Abeta-induced blood-brain barrier damage through activation of endothelial MMP9. J Neurochem. (2017) 142:464–77. 10.1111/jnc.14068 28488764

[99] Kim GS Yang L Zhang G Zhao H Selim M McCullough LD. Critical role of sphingosine-1-phosphate receptor-2 in the disruption of cerebrovascular integrity in experimental stroke. Nat Commun. (2015) 6:7893. 10.1038/ncomms8893 26243335 PMC4587559

[100] Ebrahem Q Chaurasia SS Vasanji A Qi JH Klenotic PA Cutler A. Cross-talk between vascular endothelial growth factor and matrix metalloproteinases in the induction of neovascularization *in vivo*. Am J Pathol. (2010) 176:496–503. 10.2353/ajpath.2010.080642 19948826 PMC2797907

[101] Lobb RJ Lima LG Moller A. Exosomes: Key mediators of metastasis and pre-metastatic niche formation. Semin Cell Dev Biol. (2017) 67:3–10. 10.1016/j.semcdb.2017.01.004 28077297

[102] Ahn GO Brown JM Matrix metalloproteinase-9 is required for tumor vasculogenesis but not for angiogenesis: role of bone marrow-derived myelomonocytic cells. Cancer Cell (2008) 13:193–205. 10.1016/j.ccr.2007.11.032 18328424 PMC2967441

[103] Tjiu JW Chen JS Shun CT Lin SJ Liao YH Chu CY. Tumor-associated macrophage-induced invasion and angiogenesis of human basal cell carcinoma cells by cyclooxygenase-2 induction. J Invest Dermatol. (2009) 129:1016–25. 10.1038/jid.2008.310 18843292

[104] Liu Y Gu Y Han Y Zhang Q Jiang Z Zhang X. Tumor exosomal RNAs promote lung pre-metastatic niche formation by activating alveolar epithelial TLR3 to recruit neutrophils. Cancer Cell (2016) 30:243–56. 10.1016/j.ccell.2016.06.021 27505671

[105] Li K Chen Y Li A Tan C Liu X. Exosomes play roles in sequential processes of tumor metastasis. Int J Cancer (2018). 10.1002/ijc.31774. [Epub ahead of print]. 30155891

[106] Zoller M. Janus-faced myeloid-derived suppressor cell exosomes for the good and the bad in cancer and autoimmune disease. Front Immunol. (2018) 9:137. 10.3389/fimmu.2018.00137 29456536 PMC5801414

[107] Kosaka N Iguchi H Hagiwara K Yoshioka Y Takeshita F Ochiya T. Neutral sphingomyelinase 2 (nSMase2)-dependent exosomal transfer of angiogenic microRNAs regulate cancer cell metastasis. J Biol Chem. (2013) 288:10849–59. 10.1074/jbc.M112.446831 23439645 PMC3624465

[108] Noman MZ Janji B Hu S Wu JC Martelli F Bronte V. Tumor-promoting effects of myeloid-derived suppressor cells are potentiated by hypoxia-induced expression of miR-210. Cancer Res. (2015) 75:3771–87. 10.1158/0008-5472.can-15-0405 26206559

[109] Walsh JE Clark AM Day TA Gillespie MB Young MR. Use of alpha,25-dihydroxyvitamin D3 treatment to stimulate immune infiltration into head and neck squamous cell carcinoma. Hum Immunol. (2010) 71:659–65. 10.1016/j.humimm.2010.04.008 20438786 PMC3337687

[110] Kusmartsev S Cheng F Yu B Nefedova Y Sotomayor E Lush R. All-trans-retinoic acid eliminates immature myeloid cells from tumor-bearing mice and improves the effect of vaccination. Cancer Res (2003) 63:4441–9. 12907617

[111] Kusmartsev S Su Z Heiser A Dannull J Eruslanov E Kubler H. Reversal of myeloid cell-mediated immunosuppression in patients with metastatic renal cell carcinoma. Clin Cancer Res. (2008) 14:8270–8. 10.1158/1078-0432.ccr-08-0165 19088044

[112] Bruns H Bottcher M Qorraj M Fabri M Jitschin S Dindorf J. CLL-cell-mediated MDSC induction by exosomal miR-155 transfer is disrupted by vitamin D. Leukemia (2017) 31:985–88. 10.1038/leu.2016.378 28008175

[113] Veltman JD Lambers ME van Nimwegen M Hendriks RW Hoogsteden HC Hegmans JP. Zoledronic acid impairs myeloid differentiation to tumour-associated macrophages in mesothelioma. Br J Cancer (2010) 103:629–41. 10.1038/sj.bjc.6605814 20664588 PMC2938257

[114] Zhuang J Zhang J Lwin ST Edwards JR Edwards CM Mundy GR. Osteoclasts in multiple myeloma are derived from Gr-1+CD11b+myeloid-derived suppressor cells. PLoS ONE (2012) 7:e48871. 10.1371/journal.pone.0048871 23173040 PMC3500251

[115] Porembka MR Mitchem JB Belt BA Hsieh CS Lee HM Herndon J. Pancreatic adenocarcinoma induces bone marrow mobilization of myeloid-derived suppressor cells which promote primary tumor growth. Cancer Immunol Immunother. (2012) 61:1373–85. 10.1007/s00262-011-1178-0 22215137 PMC3697836

[116] Melani C Sangaletti S Barazzetta FM Werb Z Colombo MP. Amino-biphosphonate-mediated MMP-9 inhibition breaks the tumor-bone marrow axis responsible for myeloid-derived suppressor cell expansion and macrophage infiltration in tumor stroma. Cancer Res. (2007) 67:11438–46. 10.1158/0008-5472.can-07-1882 18056472 PMC2646404

[117] Priceman SJ Sung JL Shaposhnik Z Burton JB Torres-Collado AX Moughon DL. Targeting distinct tumor-infiltrating myeloid cells by inhibiting CSF-1 receptor: combating tumor evasion of antiangiogenic therapy. Blood (2010) 115:1461–71. 10.1182/blood-2009-08-237412 20008303 PMC2826767

[118] Zhang Y Liu Q Zhang M Yu Y Liu X Cao X. Fas signal promotes lung cancer growth by recruiting myeloid-derived suppressor cells via cancer cell-derived PGE2. J Immunol. (2009) 182:3801–8. 10.4049/jimmunol.0801548 19265159

[119] Hurez V Daniel BJ Sun L Liu AJ Ludwig SM Kious MJ. Mitigating age-related immune dysfunction heightens the efficacy of tumor immunotherapy in aged mice. Cancer Res. (2012) 72:2089–99. 10.1158/0008-5472.can-11-3019 22496463 PMC3328641

[120] Condamine T Kumar V Ramachandran IR Youn JI Celis E Finnberg N. ER stress regulates myeloid-derived suppressor cell fate through TRAIL-R-mediated apoptosis. J Clin Invest. (2014) 124:2626–39. 10.1172/jci74056 24789911 PMC4038578

[121] Dominguez GA Condamine T Mony S Hashimoto A Wang F Liu Q. Selective targeting of myeloid-derived suppressor cells in cancer patients using DS-8273a, an agonistic TRAIL-R2 antibody. Clin Cancer Res. (2017) 23:2942–50. 10.1158/1078-0432.ccr-16-1784 27965309 PMC5468499

[122] Eksioglu EA Chen X Heider KH Rueter B McGraw KL Basiorka AA. Novel therapeutic approach to improve hematopoiesis in low risk MDS by targeting MDSCs with the Fc-engineered CD33 antibody BI 836858. Leukemia (2017) 31:2172–80. 10.1038/leu.2017.21 28096534 PMC5552472

[123] Garton AJ Seibel S Lopresti-Morrow L Crew L Janson N Mandiyan S. Anti-KIT monoclonal antibody treatment enhances the antitumor activity of immune checkpoint inhibitors by reversing tumor-induced immunosuppression. Mol Cancer Ther. (2017) 16:671–80. 10.1158/1535-7163.mct-16-0676 28138031

[124] Cappello P Tonoli E Curto R Giordano D Giovarelli M Novelli F. Anti-alpha-enolase antibody limits the invasion of myeloid-derived suppressor cells and attenuates their restraining effector T cell response. Oncoimmunology (2016) 5:e1112940. 10.1080/2162402x.2015.1112940 27467915 PMC4910736

[125] Kobayashi M Chung JS Beg M Arriaga Y Verma U Courtney K. Blocking monocytic myeloid-derived suppressor cell function via anti-DC-HIL/GPNMB antibody restores the *in vitro* integrity of t cells from cancer patients. Clin Cancer Res. (2018) 25:828–38. 10.1158/1078-0432.ccr-18-0330 30049749 PMC7315386

[126] Gajos-Michniewicz A Duechler M Czyz M. MiRNA in melanoma-derived exosomes. Cancer Lett. (2014) 347:29–37. 10.1016/j.canlet.2014.02.004 24513178

[127] Wang Y Tian J Tang X Rui K Tian X Ma J. Exosomes released by granulocytic myeloid-derived suppressor cells attenuate DSS-induced colitis in mice. Oncotarget (2016) 7:15356–68. 10.18632/oncotarget.7324 26885611 PMC4941246

[128] Olmeda D Cerezo-Wallis D Riveiro-Falkenbach E Pennacchi PC Contreras-Alcalde M Ibarz N. Whole-body imaging of lymphovascular niches identifies pre-metastatic roles of midkine. Nature (2017) 546:676–80. 10.1038/nature22977 28658220 PMC6005659

[129] Cox TR Rumney RMH Schoof EM Perryman L Hoye AM Agrawal A. The hypoxic cancer secretome induces pre-metastatic bone lesions through lysyl oxidase. Nature (2015) 522:106–10. 10.1038/nature14492 26017313 PMC4961239

[130] Ugel S Peranzoni E Desantis G Chioda M Walter S Weinschenk T. Immune tolerance to tumor antigens occurs in a specialized environment of the spleen. Cell Rep. (2012) 2:628–39. 10.1016/j.celrep.2012.08.006 22959433

[131] Anani W Shurin MR. Targeting myeloid-derived suppressor cells in cancer. Adv Exp Med Biol. (2017) 1036:105–28. 10.1007/978-3-319-67577-0_8 29275468

[132] Najjar YG Finke JH. Clinical perspectives on targeting of myeloid derived suppressor cells in the treatment of cancer. Front Oncol. (2013) 3:49. 10.3389/fonc.2013.00049 23508517 PMC3597982

[133] Kulbersh JS Day TA Gillespie MB Young MR. 1alpha,25-Dihydroxyvitamin D(3) to skew intratumoral levels of immune inhibitory CD34(+) progenitor cells into dendritic cells. Otolaryngol Head Neck Surg. (2009) 140:235–40. 10.1016/j.otohns.2008.11.011 19201295 PMC3337726

[134] Zhang H Maric I DiPrima MJ Khan J Orentas RJ Kaplan RN. Fibrocytes represent a novel MDSC subset circulating in patients with metastatic cancer. Blood (2013) 122:1105–13. 10.1182/blood-2012-08-449413 23757729 PMC3744987

[135] Hengesbach LM Hoag KA. Physiological concentrations of retinoic acid favor myeloid dendritic cell development over granulocyte development in cultures of bone marrow cells from mice. J Nutr. (2004) 134:2653–9. 10.1093/jn/134.10.2653 15465762

[136] Li L Zhang J Diao W Wang D Wei Y Zhang CY. MicroRNA-155 and MicroRNA-21 promote the expansion of functional myeloid-derived suppressor cells. J Immunol. (2014) 192:1034–43. 10.4049/jimmunol.1301309 24391219

[137] Martire-Greco D Rodriguez-Rodrigues N Castillo LA Vecchione MB de Campos-Nebel M Cordoba Moreno M. Novel use of all-trans-retinoic acid in a model of lipopolysaccharide-immunosuppression to decrease the generation of myeloid-derived suppressor cells by reducing the proliferation of CD34+ precursor cells. Shock (2017) 48:94–103. 10.1097/shk.0000000000000812 27922552

